# A Promptable 3D-CT Foundation Model-Based Approach for Pulmonary Embolism

**DOI:** 10.1007/s00270-026-04488-2

**Published:** 2026-06-02

**Authors:** K. Le Floch, E. Ferreres, J. Khlaut, A. Prat, L. Alberge, L. Machado, D. Tordjman, X. Guerra, P. Hérent, P. Manceron, M. Sapoval, T. Boeken

**Affiliations:** 1 Department of Vascular and Oncological interventional Radiology, Hôpital Européen Georges Pompidou, HeKA PRAIRIE-PSAI, INRIA Paris, Paris, France; 2 Raidium, Paris, France; 3 Department of Radiology, Beaujon Hospital, Clichy, France; 4 Department of Radiology, Saint-Denis, France

**Keywords:** Artificial intelligence, Foundation models, Pulmonary embolism, Interactive segmentation, Blood clot volume, Thrombotic burden, Risk stratification

## Abstract

**Purpose:**

Blood clot volume (BCV), defined as the total three-dimensional (3D) volume of the thrombus on computed tomography angiography (CTA), is an objective biomarker of pulmonary embolism (PE) severity whose clinical use is limited by time-consuming manual segmentation. This study evaluates ClotIA (Clot Interventional AI), a foundation model (FM)-based approach designed for rapid and interactive clot segmentation in PE.

**Materials and Methods:**

RAPSv2, a foundation model derived from SAM2, was fine-tuned on a stratified sample of 309 patients from the RSPECT dataset (2020). Segmentation performance was evaluated using the Dice similarity coefficient (DSC) and compared to that of nnUNet (no-new-Net). The predicted BCV was correlated with imaging biomarkers of PE severity.

**Results:**

ClotIA achieved a mean DSC of 0.83 ± 0.06 after guided refinement, compared to 0.79 ± 0.10 at baseline (*p* < 0.001) and 0.81 ± 0.13 for nnUNet (*p* < 0.001). The predicted BCV showed strong agreement with the reference volume (r = 0.995; mean bias + 0.12 mL) and was significantly correlated with RV/LV diameter ratio (r = 0.62, *p* < 0.001) and RV/LV volume ratio (r = 0.68, *p* < 0.001).

**Conclusion:**

ClotIA enables rapid and reproducible 3D quantification of pulmonary embolism thrombi, correlating with established severity markers and providing the necessary basis for translating emerging biomarkers into clinical practice.

**Graphical Abstract:**

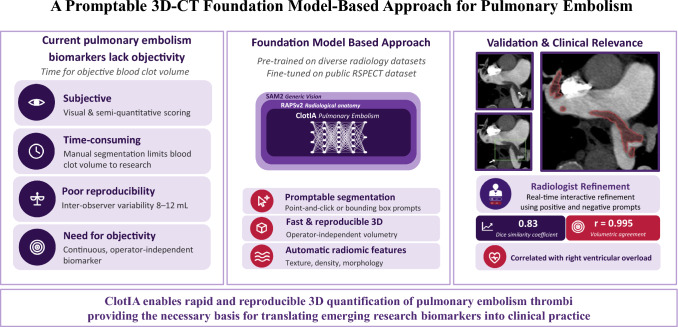

## Introduction

Pulmonary embolism (PE) is the third leading cause of cardiovascular mortality worldwide [1, 2]. Management is based on risk stratification: most patients receive anticoagulant therapy, a high-risk subgroup (3–4%) requires immediate reperfusion, and an intermediate-risk group (10–15%) may benefit from early catheter-directed thrombolysis (CDT) before the onset of hemodynamic collapse [3]. Systemic thrombolysis (the first-line treatment for high-risk PE) is associated with a 9–12% rate of intracranial haemorrhage and fails in approximately 20–30% of intermediate-to-high-risk patients, which explains the growing interest in catheter guided reperfusion therapies [4–6].

Clinical decision-making in cases of PE relies on clinical scores, cardiac biomarkers, and imaging. Current imaging biomarkers are visual and semi-quantitative, and their reproducibility limited. Blood clot volume (BCV), defined as the total 3D volume of the thrombus (in mL) derived from a CTPA, provides a continuous and objective measure for which previous studies have shown that a higher BCV correlates with signs of right ventricular (RV) overload and is associated with a higher incidence of right ventricular dysfunction. Furthermore, total embolic volume has been identified as an independent predictor of impending shock in certain cohorts [7, 8]. Its hemodynamic significance depends on both the patient’s pulmonary vascular reserve and the location of the thrombus. Recent studies further suggest that the radiomic characteristics of thrombi have independent prognostic value and could improve risk stratification beyond volume alone [9–12]. Despite this potential, BCV quantification remains confined to research due to the time-consuming nature of manual segmentation [13]. Moreover, the recent publication of the 2026 guidelines on acute PE underscores the urgent need for more accurate quantitative tools to guide therapeutic decision-making [14].

The rapid evolution of artificial intelligence (AI), particularly foundation models (FMs) in radiology, has the potential to address this urgent need and offer a fundamentally different approach to overcoming this limitation [15, 16]. Unlike traditional task-specific AI, FMs are pre-trained on massive datasets, enabling them to generalize across anatomical regions, imaging protocols, and clinical tasks with minimal fine-tuning [17–19]. Those models allow for a true understanding of the image within a unified representation space, enabling the simultaneous capture of the thrombus’s complete radiomic signature.

We developed ClotIA (Clot Interventional AI), a 3D-CT FM-based approach, optimized for thrombus segmentation radiologist-guided, with automatic feature extraction in patients with PE. The objective of this study is to evaluate and report the key performances of ClotIA on a publicly available dataset.

## Materials and Methods

This retrospective study followed the recommendations of the I-CARE group [20].

### Study Design and Dataset

The RSPECT dataset comprises 12,195 computed tomography pulmonary angiograms (CTPA) from the 2020 RSNA PE Detection Challenge, each of which was labelled by RSNA-affiliated radiologists to indicate the presence of a pulmonary embolism (PE), the location of the thrombus, the right ventricle (RV)/left ventricle (LV) ratio, and chronicity without segmentation masks [21]. After excluding 298 examinations due to artifacts or insufficient contrast, 11,898 examinations remained eligible. Using this existing labeling of PE-positive patients, case selection and manual segmentation were performed by two junior radiologists (residents) and two senior interventional radiologists with 10 and 20 years of experience respectively. We applied stratified random sampling to select 309 PE-positive patients, ensuring a balanced representation of proximal and segmental thrombus distributions across a wide range of embolic loads, using the thrombus location labels provided by the RSNA. Each case was then manually segmented for the entire pulmonary artery tree, following a standardized annotation protocol. Disagreements were resolved by consensus, constituting the ground truth reference. The dataset was divided into training (n = 210), validation (n = 42), and test (n = 57) sets.

This retrospective study was based exclusively on public, anonymised data from the RSPECT dataset (RSNA Pulmonary Embolism CT Challenge, 2020). In accordance with the institution’s policy, no formal ethical approval was required. No patient-identifying information was utilized.

### Model Development

**Fig. 1:**
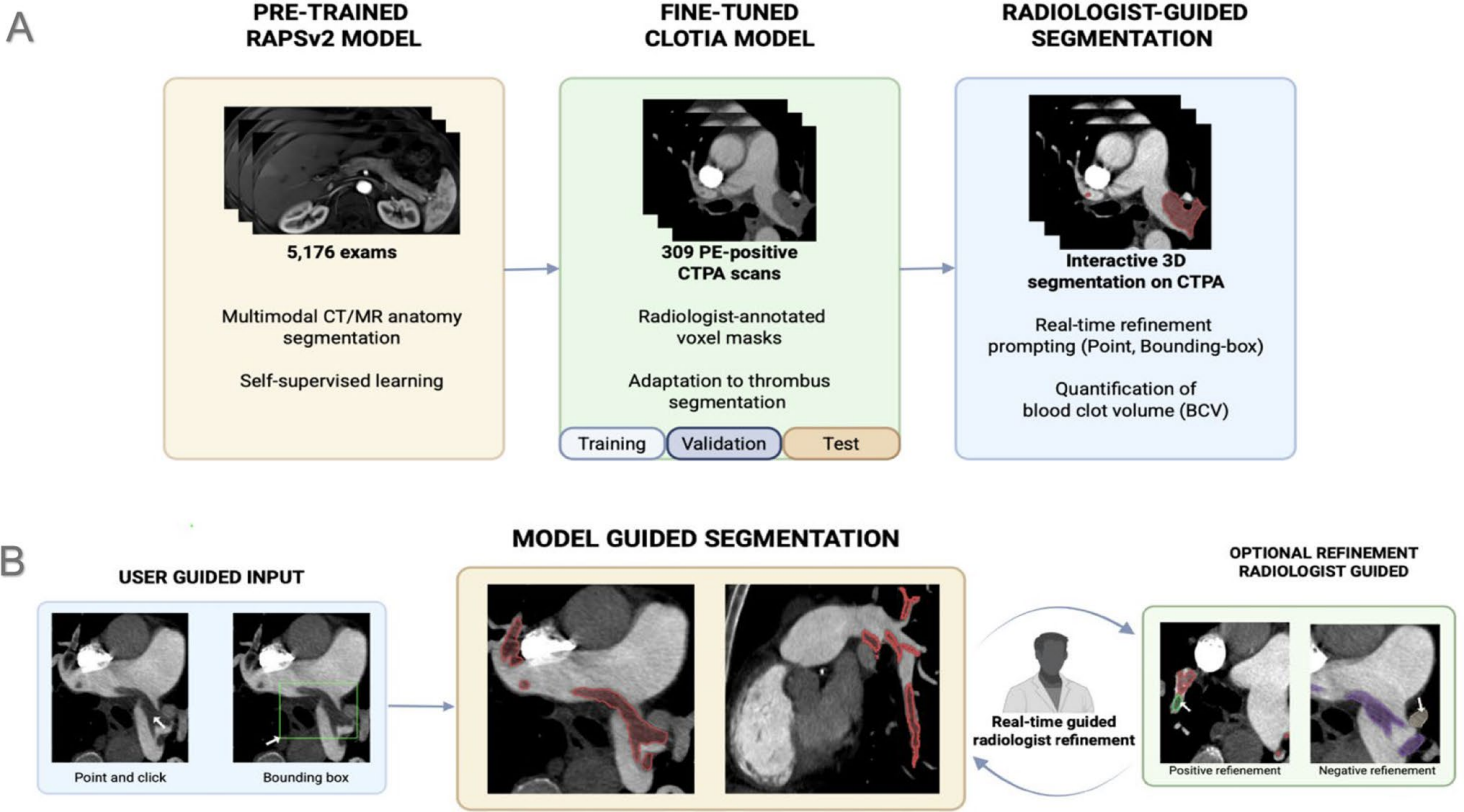
**A** Model pre-training and fine-tuning pipeline. RAPSv2 was initially pre-trained on a diverse set of general anatomy datasets (5,176 exams), including CT and MRI scans from multiple anatomical regions. This multi-modal pre-training enhances the model’s generalization capability across different imaging modalities and protocols. A subset of the RSNA Pulmonary Embolism CT (RSPECT) dataset, comprising 309 high-quality CTPA scans from patients annotated for thrombus segmentation for ClotIA fine-tuning. **B** Pipeline of ClotIA multimodal interaction workflow. Radiologist-provided input, via point-and-click or bounding boxes on the CTPA images, is integrated into the model-guided segmentation, producing the initial thrombus segmentation boundaries (indicated by red overlays). The radiologist can optionally refine the segmentation, allowing positive (additive) or negative (subtractive) adjustments to refine the segmentation for accurate assessment of pulmonary embolism

ClotIA was developed by fine-tuning RAPSv2, a 3D-CT FM model for medical imaging that had been pre-trained on 5,176 diverse radiological examinations including CT scans and MRIs covering multiple anatomical regions and derived from the SAM2 architecture, which was itself trained on over a billion masks from the SA-1B dataset [22]. Interactive 3D segmentation is performed using two input methods: “point-and-click,” in which the radiologist places one or more starting points directly on the thrombus in any imaging plane, and the “selection box,” in which a rectangular region of interest is outlined around the target structure. These two input methods are propagated throughout the 3D volume to generate an initial segmentation mask. The radiologist can then refine this mask iteratively using positive or negative prompts, with each correction updating the 3D segmentation in real time. To simulate this interactive refinement during fine-tuning, synthetic prompts were generated iteratively from error maps calculated between the current prediction and the ground truth, thereby mimicking a radiologist’s corrective behaviour (Fig. 1).

### Evaluation Metrics and Statistical Analysis

Statistical analyses were performed using R (version 4.3.2) and GraphPad Prism (version 11; GraphPad Software, San Diego, California, USA). The primary evaluation criteria were segmentation accuracy, measured by the Dice Similarity Coefficient (DSC), which quantifies spatial overlap between automated and expert reference masks (range: 0 [no overlap] to 1 [perfect overlap]) [23]. Segmentation performance was reported as mean ± standard deviation. Comparisons of (DSC) between models and configurations were performed using the Wilcoxon signed-rank test. Analyses stratified by thrombus volume quartiles were assessed using the Kruskal–Wallis test. Volumetric agreement was evaluated using linear regression (R^2^), Spearman’s correlation, and Bland–Altman analysis (with 95% limits of agreement). Correlations between predicted blood clot volume and imaging biomarkers of right ventricular overload were assessed using Spearman’s correlation. All statistical tests were two-sided, and a *p*-value < 0.05 was considered statistically significant.

## Results

### Segmentation Performance and Volumetric Accuracy

**Fig. 2:**
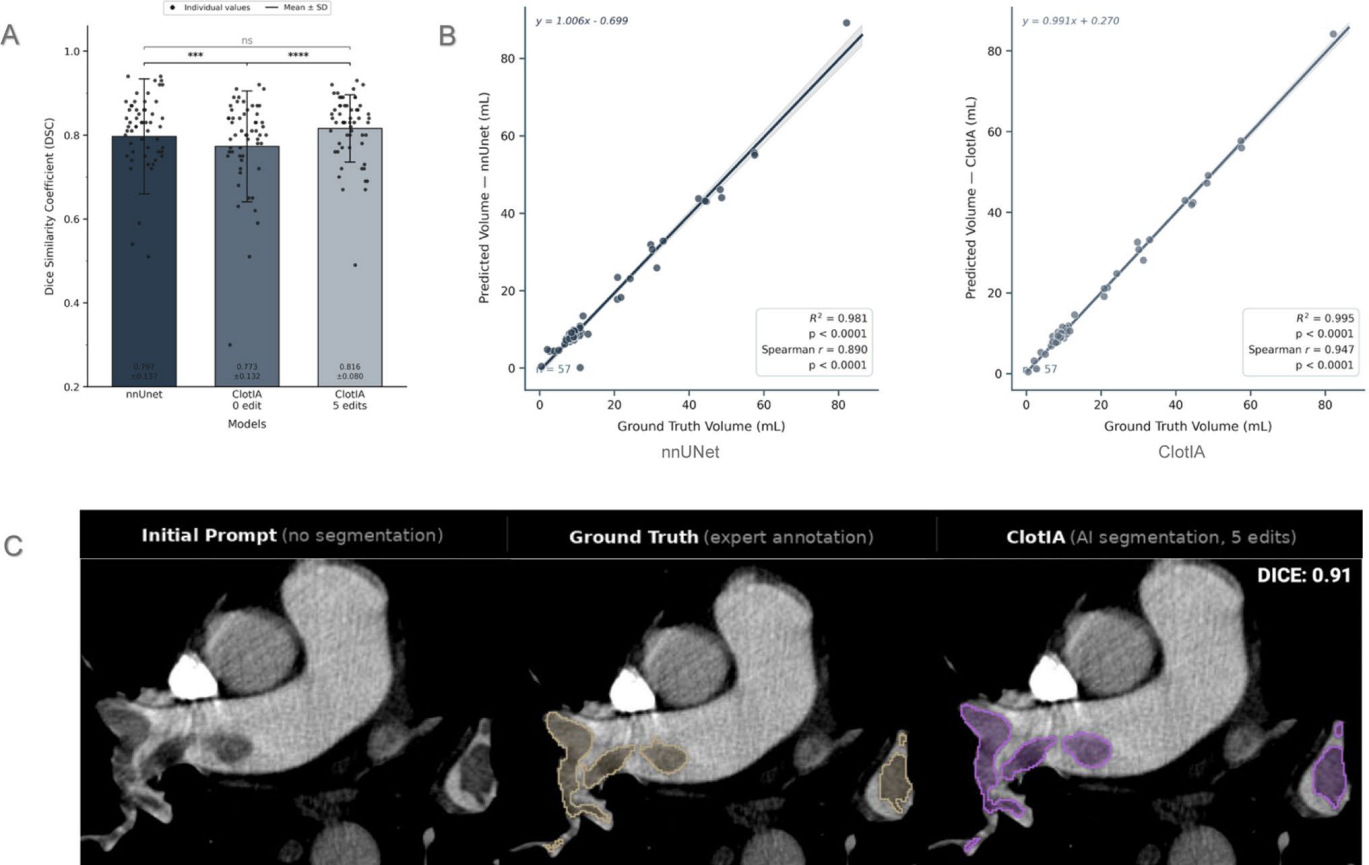
Overall segmentation performance and agreement metrics for pulmonary embolism models. **A** Scatter plot showing the distributions of the Dice similarity coefficient (DSC) for the nnUNet and ClotIA (0 and 5 edits) models in all patients tested (n = 57). Individual data points represent DSC values for each CTPA scan. **B** Linear correlation between predicted thrombus volumes and reference volumes for the ClotIA (5 edits). The solid line represents the linear regression and the shadow zone the 95% confidence interval. **C** Qualitative cases of ClotIA segmentation performance. CTPA slices illustrating the initial visual prompt (left), expert reference annotation (middle), and final ClotIA prediction (right) with DSC performance (top right)

The mean thrombus volume was 38.9 ± 7.4 mL (range: 2.0–82.0 mL). At baseline (0 edit), ClotIA achieved a mean DSC of 0.793 ± 0.104, which improved significantly to 0.828 ± 0.063 after five guided edits (*p* < 0.001). nnUNet reached 0.809 ± 0.134 with no significant difference compared to ClotIA 5 edits in terms of mean DSC (*p* = 0.795), but with significantly lower accuracy (Table 1, Fig. 2).

**Table 1:** Dice similarity coefficient by model and thrombus volume quartile. DSC values are presented for nnUNet and ClotIA (0 and 5 editing iterations) across the overall cohort (n = 57) and stratified by thrombus volume quartile (n = 13 per quartile)

	nnUNet	ClotIA 0 edit	ClotIA 5 edits
*Overall DSC (n* = *57)*			
Mean ± SD	0.809 ± 0.134	0.793 ± 0.104	0.828 ± 0.063
Median	0.830	0.810	0.835
IQR	0.778–0.870	0.768–0.853	0.800–0.870
Range	0.010–0.940	0.300–0.920	0.670–0.930
*Pairwise comparisons*			
ClotIA 0 edit vs ClotIA 5 edits		*p*-value	< 0.001
*DSC by Thrombus Volume Quartile*			
Volume quartile	Mean ± SD Median	Mean ± SD Median	Mean ± SD Median
Q1 (< 8.4 mL)	0.785 ± 0.047 0.790	0.757 ± 0.071 0.760	0.785 ± 0.067 0.780
Q2 (8.4–9.5 mL)	0.826 ± 0.042 0.830	0.825 ± 0.035 0.810	0.835 ± 0.032 0.830
Q3 (9.5–24.1 mL)	0.722 ± 0.229 0.800	0.745 ± 0.143 0.780	0.806 ± 0.064 0.830
Q4 (> 24.1 mL)	0.904 ± 0.028 0.900	0.847 ± 0.105 0.870	0.885 ± 0.036 0.890

DSC, Dice Similarity Coefficient; IQR, interquartile range; SD, standard deviation

**Fig. 3:**
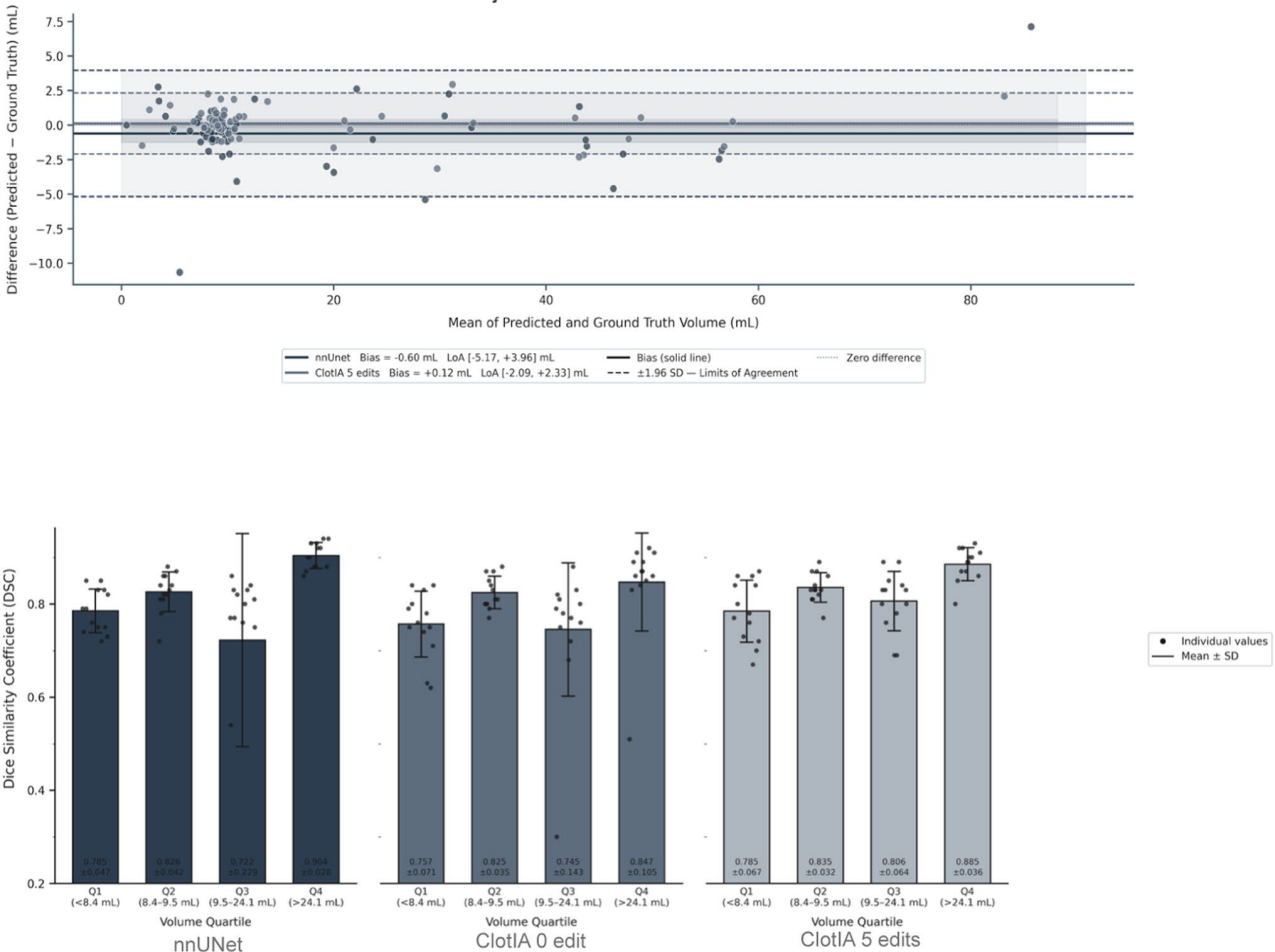
Bland–Altman plot of volumetric agreement and DSC, stratified by quartile of thrombus volume. (Top) Bland–Altman plot comparing predicted thrombus volume to the expert-established reference for nnUNet (dark line) and the modifications made by ClotIA 5 (light line). Solid lines indicate model-specific bias; dashed lines indicate agreement limits ± 1.96 standard deviations; the dotted line indicates a zero difference. (Bottom) DSC stratified by thrombotic volume quartile (Q1 < 8.4 mL, Q2 8.4–9.5 mL, Q3 9.5–24.1 mL, Q4 > 24.1 ml; n = 13 per quartile) for nnUNet **A**, ClotIA 0 modifications **B**, and ClotIA 5 modifications **C**. Bars represent the mean ± standard deviation, with individual data points overlaid

Volumetric agreement was significant for ClotIA 5 edits (R^2^ = 0.995, Spearman’s r = 0.947; *p* < 0.001), with a mean bias of + 0.12 mL and narrow Bland–Altman limits of agreement (LoA: − 2.09 to + 2.33 mL; total: 4.42 mL), well below the inter-observer variability typical of manual segmentation (8–12 mL) [25]. In contrast, nnUNet showed a borderline negative bias of − 0.60 mL and significantly wider limits of agreement (LoA: − 5.17 to + 3.96 mL; total: 9.13 mL) (Table 2, Fig. 3).

**Table 2:** Bland–Altman agreement analysis: nnUNet and ClotIA (5 modifications) compared to the actual thrombus volume. A Bland–Altman analysis was performed to assess volumetric agreement between each segmentation model and the expert-defined reference (n = 57). Bias corresponds to the mean difference between the predicted volume and the actual volume (Predicted volume; Actual volume, in ml). Limits of agreement (LoA) were defined as bias deviations. The 95% confidence interval for the bias was calculated using the t-distribution

Parameter	nnUNet	ClotIA 5 edits
*Bias*		
Mean bias (mL)	− 0.603	+ 0.123
95% CI of bias (mL)	[− 1.221, + 0.015]	[− 0.176, + 0.423]
Bias significance	*p* = 0.05	*p* > .05
*Precision*		
SD of differences (mL)	2.330	1.128
Upper LoA (mL)	+ 3.963	+ 2.334
Lower LoA (mL)	− 5.169	− 2.087
Total LoA (mL)	9.132	4.421

### Correlation with Imaging Biomarkers Reflecting the Severity of PE

**Fig. 4:**
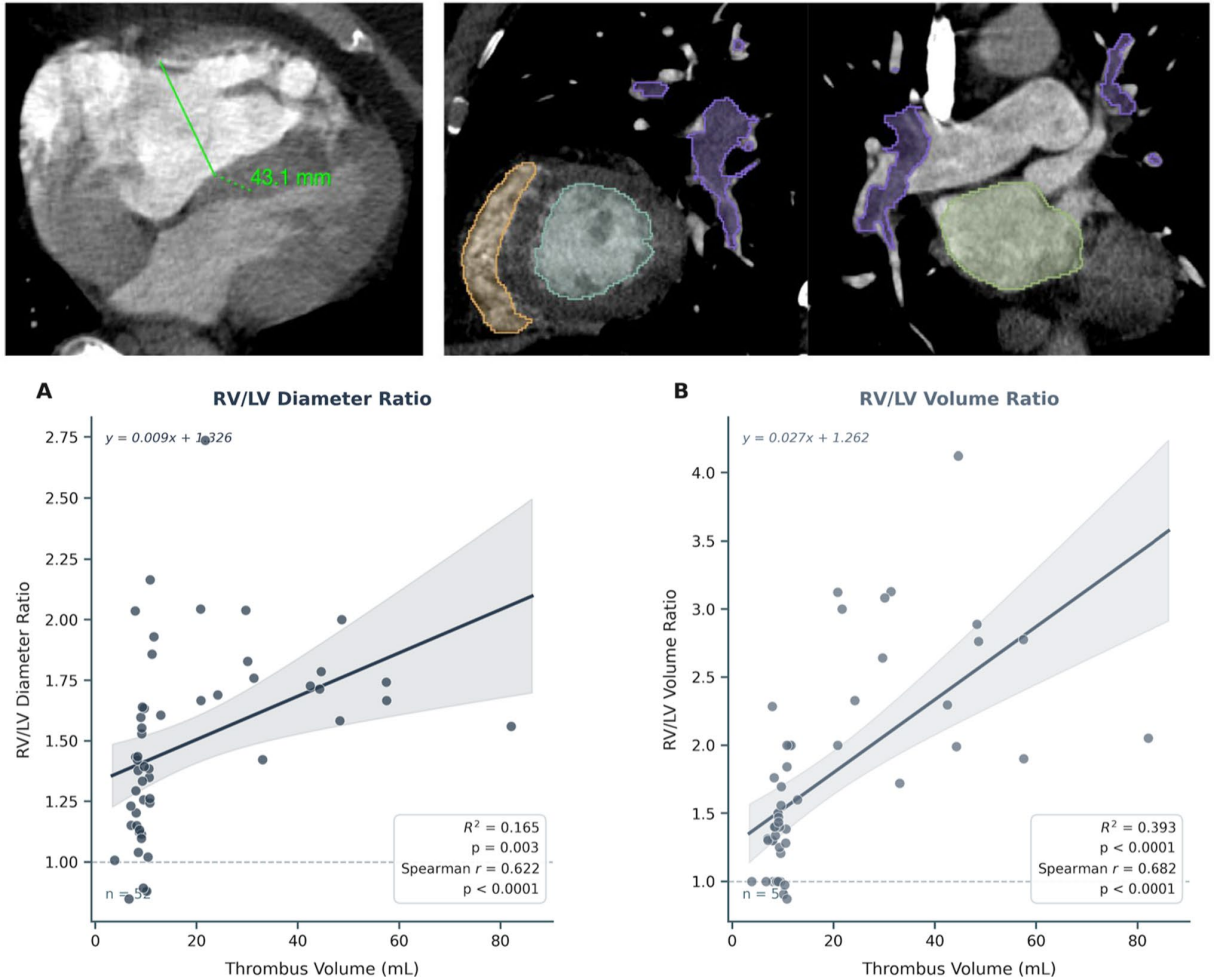
Imaging biomarkers of pulmonary embolism severity stratified by thrombus volume quartiles. **A** Illustrative CTPA scan images showing diameter measurements (left, right ventricle to left ventricle diameter ratio RV/LV with calibre annotation) and volumetric automatic segmentations with the RadSam Model for general anatomy (right: RV in yellow, LV in cyan, right atrium in purple, and left atrium in green, with isolated thrombi in blue. **B** Quantitative correlation between thrombus volume and right heart overload markers. Scatter plots showing the positive correlation between segmented thrombus volume predicted by ClotIA (5-edits) and right ventricular overload markers

ClotIA-predicted BCV showed a significant correlation with all markers of right ventricular overload: RV/LV diameter ratio (Spearman’s r = 0.62, p < 0.001), RV/LV volume ratio (r = 0.68, *p* < 0.001), RV volume (r = 0.42, *p* < 0.01), and RA volume (r = 0.29, *p* = 0.036). In contrast, LV volume (r = 0.02, *p* = 0.91) and LA volume (r = 0.03, *p* = 0.83) showed no association (Table 3, Fig. 4).

**Table 3:** Spearman’s correlations between cardiac severity biomarkers and thrombus volume. Spearman’s correlation coefficients (r) and corresponding *p*-values are presented for six cardiac biomarkers relative to the expert-defined reference and the thrombus volume predicted by ClotIA (5 edits; n = 57). Mean ± standard deviation are provided for each biomarker

Severity biomarker	Mean ± SD	Ground truth volume *r (p-value)*	ClotIA predicted volume *r (p-value)*
RV/LV Diameter ratio	1.49 ± 0.37	0.66 (< 0.001)	0.62 (< 0.001)
RV Volume (mL)	169.27 ± 69.10	0.42 (< 0.01)	0.42 (< 0.01)
LV Volume (mL)	89.56 ± 31.92	0.00 (0.97)	0.02 (0.91)
RA Volume (mL)	100.73 ± 53.97	0.30 (0.034)	0.29 (0.036)
LA Volume (mL)	60.12 ± 24.30	0.05 (0.72)	0.03 (0.83)
RV/LV Volume ratio	1.76 ± 0.73	0.69 (< 0.001)	0.68 (< 0.001)

LA, left atrium; LV, left ventricle; RA, right atrium; RV, right ventricle; SD, standard deviation

## Discussion

Despite the recognized clinical relevance of BCV, its routine use in the standard management of PE remains out of reach because there is no universally available software capable of providing the clot segmentation. The semi-manual segmentation process is time-consuming, operator-dependent, and offers low reproducibility across centers, which limits thrombus volumetry to isolated research protocols.

ClotIA was developed to address this gap, demonstrating that accurate, rapid, and reproducible 3D thrombus quantification is achievable on a large scale. ClotIA achieves a mean DSC of 0.828 after five edits, significantly higher than the baseline (*p* < 0.001) and more accurate than nnUNet. This precision is well below the typical inter-observer variability of manual segmentation (8–12 mL), making the BCV derived from ClotIA a reproducible, operator-independent biomarker and a reliable data point for future analyses [7].

While ClotIA provides an accessible and objective quantification of the blood clot volume (BCV), it is important to recognize that this biomarker alone does not capture the full complexity of the condition. More specifically, BCV does not account for the anatomical location or spatial distribution of the thrombus, nor does it reflect the patient’s underlying pulmonary vascular reserve.

It is essential to recognize these limitations, as the hemodynamic impact of a given volume remains intrinsically linked to these unmeasured variables and the patient’s specific cardiopulmonary adaptation. In this way, the clinical value of BCV is fully realized when it serves as a complementary tool within a broader multiparametric assessment, ensuring that objective volumetric data are interpreted with consideration of the clinical and hemodynamic context. Previous studies support this integrative approach by showing that combining thrombus volume with cardiac imaging parameters and markers such as troponin I or NT-proBNP significantly improves the prediction of right ventricular dysfunction compared to the use of a single parameter [13, 26, 27], similarly to the integration of these biomarkers into existing scoring frameworks like Qanadli score, allows for better risk stratification [28]. This is precisely where the strength of a foundation model-based approach lies within the inherent limitations of simple volumetry. Unlike traditional AI specialized in specific tasks, FMs benefit from a unified representation space and a deeper “understanding of the image” that allows it to see beyond the 3D extent of the clot, pay “attention” to the surrounding environment, and thus weight its outputs accordingly.

In the future, ClotIA could be integrated into a comprehensive decision-support tool for personalized interventional therapy. Previous studies using standard AI models analyzing one or more imaging parameters are moving in this direction, notably with texture analysis enabling the prediction of short-term prognosis [9], radiomic scores outperforming clinical scores in predicting adverse outcomes [10], and dual-energy CT radiomics enabling independent risk stratification [11, 12].

Several limitations should be considered. ClotIA was evaluated on a cohort of 309 patients using primarily high-quality scans with optimal contrast and minimal artifacts. In real-world emergency situations, suboptimal bolus timing or motion artifacts could affect performance. Furthermore, although we demonstrated a strong correlation between BCV and right ventricular strain, the lack of an external validation cohort and direct correlation with clinical outcomes currently limits its immediate generalizability. Future steps are already underway to address these gaps through external validation studies to confirm generalizability and assess clinical utility in therapeutic decision-making in interventional radiology.

## Conclusion

ClotIA enables rapid and reproducible 3D quantification of pulmonary embolism thrombi, correlating with established severity markers and providing the necessary basis for translating emerging biomarkers into clinical practice.

## Abbreviations

AI: Artificial intelligence
BCV: Blood clot volume
CDT: Catheter-directed thrombolysis
CT: Computed tomography
CTA: Computed tomography angiography
CTPA: Computed tomography pulmonary angiograms
DSC: Dice similarity coefficient
FM: Foundation model
I-CARE: Interventional radiology reporting standards and checklist for artificial intelligence research evaluation
LA: Left atrial
LoA: Limits of agreement
LV: Left ventricle
MRI: Magnetic resonance imaging
PE: Pulmonary embolism
RA: Right atrial
RSNA: Radiological society of north america
RSPECT: RSNA pulmonary embolism CT
RV: Right ventricle
SAM2: Segment anything model 2
